# Hacking the Cell: Network Intrusion and Exploitation by Adenovirus E1A

**DOI:** 10.1128/mBio.00390-18

**Published:** 2018-05-01

**Authors:** Cason R. King, Ali Zhang, Tanner M. Tessier, Steven F. Gameiro, Joe S. Mymryk

**Affiliations:** a Department of Microbiology & Immunology, University of Western Ontario, London, Ontario, Canada; b Department of Oncology, University of Western Ontario, London, Ontario, Canada; c London Regional Cancer Program and Lawson Health Research Institute, London, Ontario, Canada; University of Michigan-Ann Arbor; University of Texas Health Science Center at Houston

**Keywords:** chromatin, E1A, hub protein, human adenovirus, protein-protein interaction, transcription

## Abstract

As obligate intracellular parasites, viruses are dependent on their infected hosts for survival. Consequently, viruses are under enormous selective pressure to utilize available cellular components and processes to their own advantage. As most, if not all, cellular activities are regulated at some level via protein interactions, host protein interaction networks are particularly vulnerable to viral exploitation. Indeed, viral proteins frequently target highly connected “hub” proteins to “hack” the cellular network, defining the molecular basis for viral control over the host. This widespread and successful strategy of network intrusion and exploitation has evolved convergently among numerous genetically distinct viruses as a result of the endless evolutionary arms race between pathogens and hosts. Here we examine the means by which a particularly well-connected viral hub protein, human adenovirus E1A, compromises and exploits the vulnerabilities of eukaryotic protein interaction networks. Importantly, these interactions identify critical regulatory hubs in the human proteome and help define the molecular basis of their function.

## INTRODUCTION

As a consequence of the limited coding capacity of viral genomes, viral proteins that engage in rewiring cellular interaction networks are often extremely modular and multifunctional. These proteins can exist as dense assemblages of interaction motifs and domains which enable concurrent association with numerous cellular hubs (1, 2). This high level of connectivity is often accompanied by a lack of globular structure (3, 4). Instead, highly modular viral proteins contain large regions that exist in a dynamic, conformationally disordered state. These, in combination with the more conventional structured domains, provide the molecular basis for their promiscuous interaction abilities.

## MANIPULATION OF CELLULAR PROTEIN INTERACTION NETWORKS BY VIRAL PATHOGENS

The purpose of restructuring the protein interaction networks within an infected cell is to convert it to a compliant state amenable to viral replication. Given the spatial limitations of viral genomes, how a single viral protein can usurp so many pathways remains an important issue. Mechanistically, viral proteins can alter and inhibit the functions of their targets or even establish novel connections within networks not present under uninfected conditions. The remodeling of protein-protein interactions may confer radical changes to downstream cellular functions, revealing a deeper understanding of the molecular biology underlying the virus-host relationship (5).

This review focuses on how the human adenovirus (HAdV) E1A proteins invade and modify eukaryotic protein interaction networks. E1A possesses many simple and yet elegant mechanisms that allow it to drastically alter the intracellular landscape. As such, E1A exemplifies the modular and multifunctional nature of many viral proteins. The following sections describe the plethora of cellular processes affected by E1A and highlight the underlying interactions with specific host targets.

## ADENOVIRUS E1A: INVASIVE VIRAL HUB PROTEIN

Human adenoviruses are among the genetically distinct but functionally similar members of the group of small DNA tumor viruses that includes other well-studied pathogens such as human papillomavirus (HPV) and human polyomavirus (HPyV) (6, 7). Each of these viruses induces cancer in either animal or human systems, primarily by causing alterations to the host cell’s protein interaction networks (8). Each also encodes hub proteins responsible for these physiological changes. Here we focus on E1A from HAdV, the first viral gene product expressed postinfection.

E1A is essential for the HAdV replication cycle (9) and is differentially spliced into multiple isoforms. The two largest (encoding proteins of 289 and 243 amino acids [aa] in HAdV-5) are the predominant isoforms early during infection, differ only by the absence of an internal 46-aa stretch in the smaller protein, and carry out most of E1A’s known functions (10, 11). Three smaller isoforms (encoding proteins of 217, 171, and 55 aa) dominate at later times postinfection but have been less extensively characterized (12–15).

**FIG 1:**
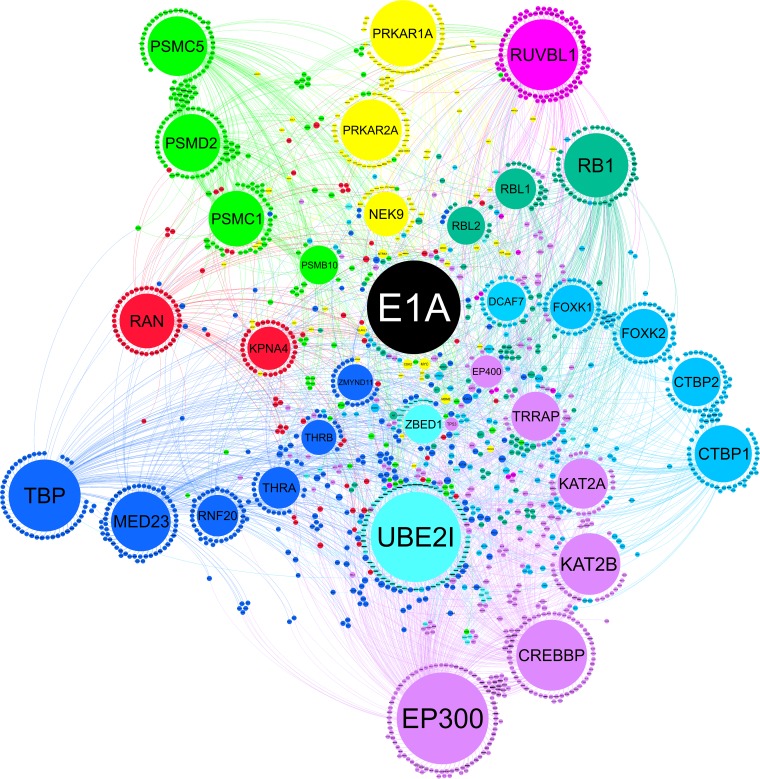
The HAdV-5 E1A interactome. A representation of the HAdV-5 E1A protein interaction network in eukaryotic cells is presented. The network was constructed using Gephi 0.9.2 with data from BioGRID build 3.4.144. E1A is placed in the center of the network, with its primary interacting partners depicted as the large circles emanating outward. The data corresponding to each primary interactor are supported by at least two peer-reviewed publications, and the size of each is proportional to the number of its own binding partners. These secondary interactors are depicted as the smaller circles, which are colored and positioned near the primary E1A interactor to which they bind. In total, 32 primary and 2,207 secondary binding partners are depicted along with the 4,087 unique edges between all depicted proteins.

E1A serves to condition the cellular environment into a state favorable for HAdV infection by interacting with many cellular regulatory proteins (16–20). In contrast to other well-studied hub proteins, E1A has no intrinsic DNA-binding or enzymatic activities; instead, it acts in a modular fashion by binding to and altering the functions of dozens of distinct cellular targets (21–23) (Fig. 1). By embedding itself deeply within the cellular protein interaction network, E1A exerts tremendous control over both viral and cellular gene expression. Indeed, based on 32 primary interactions with cellular hub proteins, HAdV-5 E1A potentially makes secondary interactions with over 2,000 other cellular targets (Fig. 1). In combination, the interplay between E1A and these primary and secondary targets represents over 4,000 unique associations, comprising a significant portion of the entire human proteome. As a consequence, E1A is able to modulate the production of other HAdV gene products, suppress innate immunity, and force the cell into the S phase of the cell cycle (24–27).

**FIG 2:**
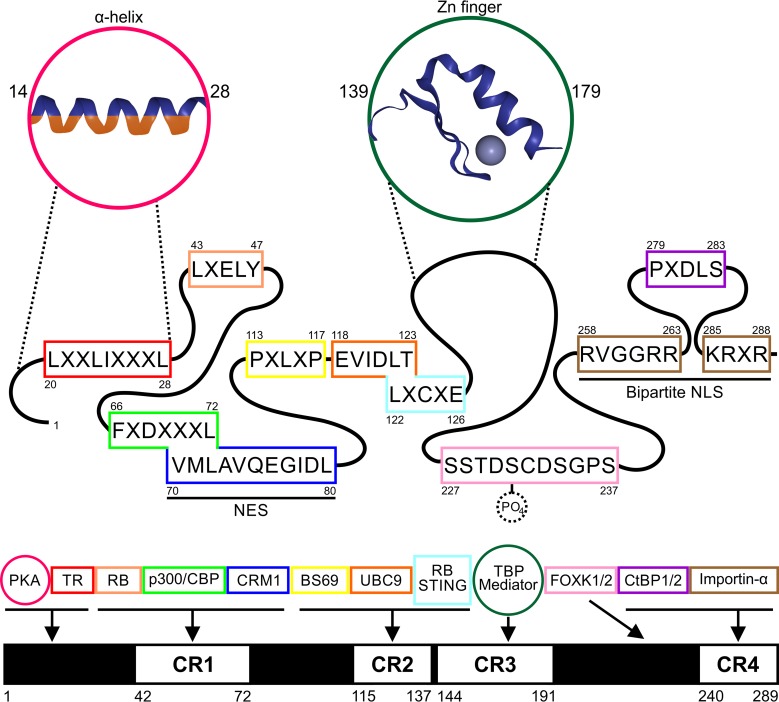
Diagrammatic representation of the protein interaction motifs of HAdV-5 E1A. Experimentally validated protein interaction motifs are displayed with their corresponding amino acid positions. These include both SLiMs (in boxes) and globular domains (in circles). Separate but overlapping SLiMs are depicted in superimposed form. Generic α-helix and zinc finger structures were adapted from entries in the Protein Data Bank (PDB) (2GLH and 2MXP) (195, 196). Notable interactions with cellular proteins conferred by these motifs are also listed and color-coded, and their associations relative to conserved regions of E1A (CR1 to CR4) are depicted.

E1A’s ability to target multiple host targets concurrently despite its small size stems from the fact that E1A is largely intrinsically disordered and contains numerous short linear interaction motifs (SLiMs) (1, 20, 28). Thus, E1A exists in a dynamic and flexible state, enabling it to sample various conformations to maximize the number of interactions using minimal coding capacity (Fig. 2). There are four regions of amino acid sequence similarity, termed conserved region 1 (CR1) through CR4, across HAdV species (29). While CR1 and CR2 of E1A bear some similarity to parts of HPV E7 and HPyV large T antigen, E1A has no known cellular ortholog (30–32). Instead, these motifs likely evolved independently as functional mimics of cellular counterparts that, presumably, provide a selective advantage for HAdV.

## INITIAL SYSTEM BREACH

Eukaryotic cells are comprised of numerous subcellular compartments and organelles, allowing more complex functionality than the prokaryotic counterparts. Trafficking and localization of proteins between distinct cellular loci comprise a carefully orchestrated process often carried out by large protein complexes (33, 34). Unsurprisingly, viruses have evolved to take advantage of this machinery to shuttle their own components between compartments within an infected cell (35–38). Like most DNA viruses, HAdV replicates within the nucleus and has evolved the means to hijack microtubule-mediated transport to deliver virions to the nuclear envelope (39, 40). Consequently, E1A synthesized during the early phase of infection must also be trafficked back into the nucleus after translation in order for the viral replication cycle to progress (41, 42). E1A is the first protein produced postinfection; it quickly begins conditioning the cell for infection by initiating the immediate burst of early viral transcription (24) and modulating the host’s transcriptional profile (25). To achieve this, E1A has evolved several ways of regulating its nuclear-cytoplasmic transport.

### Hijacking nuclear import machinery.

Passage of cellular proteins between the nucleus and cytoplasm is regulated by concerted actions of the nuclear pore complex (NPC) and soluble transport receptors known as karyopherins (importins/exportins/transportins) (43). The NPC is a molecular sieve, allowing smaller proteins to diffuse through its inner channel whereas larger proteins require an active transport mechanism provided by karyopherins and hydrolysis of RanGTP. Nuclear proteins utilizing active transport typically contain a classical nuclear localization signal (cNLS) that allows interaction with one (or more) of the seven karyopherin-α proteins (importin-α) expressed in humans (44, 45). A typical cNLS is characterized by either one (monopartite) or two (bipartite) clusters of basic amino acids, exemplified by the simian virus 40 (SV40) large T antigen (126-PKKKRKV-132) and Xenopus laevis nucleoplasmin (155-KRPAATKKAGQAKKKK-170) cNLSs, respectively (46, 47). Once bound to a cargo protein’s cNLS, importin-α interacts with karyopherin-β1 (importin-β1), allowing it to dock at the NPC and shuttle into the nucleus. Given the importance of nucleocytoplasmic transport pathways in the cellular life cycle, it is unsurprising that viruses have evolved mechanisms to commandeer them. By gaining unfettered access to and from the nuclear compartment, viral proteins such as E1A position themselves advantageously to regulate fundamental nuclear processes such as gene expression.

While E1A is known to interact with both cytoplasmic and nuclear partners, most of its targets reside in the nucleus (20) (Fig. 1). Efficient nuclear localization is required for it to carry out functions required for viral replication and cellular transformation (42, 48). To achieve this, HAdV-5 E1A interacts with cellular Qip1 (importin-α3) through a recently redefined C-terminal cNLS (49, 50). Originally described as a monopartite cNLS comprised of the sequence KRPRP, an additional stretch of basic amino acids preceding this motif is also critical for nuclear localization and interaction with Qip1. Point mutations within the major and minor binding grooves of Qip1 that prevent binding of the nucleoplasmin bipartite cNLS similarly affected E1A. This confirmed that E1A’s interaction with Qip1 was via a bipartite cNLS located between amino acid 258 and amino acid 289 (258-RVGGRRQAVECIEDLLNEPGQPLDLSCKRPRP-289) (Fig. 2). Close examination of this bipartite cNLS yielded several interesting observations. First, the linker region between basic amino acid clusters is atypically long at 21 amino acids compared to the optimal bipartite cNLS linker length, which is thought to range between 10 and 16 amino acids (45, 51). Second, within the linker is an embedded PxDLS SLiM, which confers a strong interaction with C-terminal binding protein (CtBP) (52) (Fig. 1 and 2). The presence of this motif lengthens the linker, potentially reducing the efficiency of nuclear localization (53). However, given E1A’s limited size, the tradeoff resulting from the presence of overlapping binding motifs appears to trump any reduction in nuclear import. Third, any potential loss of binding affinity due to the linker length may be compensated for by the presence of additional basic amino acids such as R258 in the upstream cluster. Notably, basic residues equivalent to R258, R262, and R263 are absolutely conserved across all known HAdV E1A proteins, supporting the idea of a critical role for these residues in nuclear import (29).

### Redefining knowledge of nonclassical nuclear import signals.

The classical nuclear import pathway is assumed to facilitate the majority of protein nuclear import; however, bioinformatic analyses of the nuclear proteome from both yeast and mouse cells revealed that over 50% of nuclear proteins do not have a predictable cNLS (54, 55). Given the predictable nature of cNLSs, these findings present interesting issues regarding what other mechanisms of nuclear import exist and what types of NLSs facilitate it.

HAdV-5 E1A is a useful molecular tool providing clues to alternative mechanisms of protein nuclear import. E1A has strategically evolved unconventional mechanisms to gain access to the nucleus. These include the previously described cNLS (50, 56) as well as at least two noncanonical NLSs located in CR1 and CR3, corresponding to aa 60 to 89 and 142 to 182, respectively (57, 58). Intriguingly, these regions show no resemblance to cNLSs and yet they are able to induce nuclear import in both yeast-based and mammalian cell-based nuclear import assays. These activities also appear to be highly conserved as they have been observed within representative HAdVs from 6 of the 7 classified species (58). Exactly how these noncanonical NLSs function remains to be fully explored. Coimmunoprecipitations (Co-IPs) suggest the N terminus (aa 1 to 82) of E1A binds both human Rch1 (importin-α1) and Qip1 (importin-α3)—two evolutionarily distinct subfamilies of importin-α (59). Results of *in vitro* binding experiments suggest that the E1A-Qip1 interaction is indirect, possibly occurring through a “piggy-back” mechanism involving one or more of E1A’s numerous other binding partners.

The amount of coding space that HAdV has devoted to ensuring nuclear transport of E1A via distinct, nonoverlapping regions illustrates the profound importance of this pathway for the virus. Interestingly, it has now emerged that nucleocytoplasmic transport of E1A is a process that can affect the subcellular localization of some of its binding targets, including protein kinase A (PKA) (described below). Additionally, dysfunctional nucleocytoplasmic transport has pathophysiological consequences, suggesting that E1A can be used to probe the molecular link between host machinery and human disease (60, 61).

## IDENTITY THEFT

Mechanisms regulating spatial and temporal control of proteins involved in cellular signaling offer an appealing target for viral manipulation. Scaffold or adapter proteins typically bind multiple members of signaling pathways and localize them to distinct subcellular loci (62–64). These highly connected hubs nucleate the formation of signalosomes that more efficiently perform pleiotropic functions in signal transduction pathways. Hijacking this part of a host’s interaction network by manipulating or mimicking cellular scaffolds potentially allows viral access to a plethora of downstream functions (1).

### Usurping a cellular scaffold.

Viral modulation of scaffold and adapter protein functions is widespread, indicative of it being a broadly beneficial strategy. HAdV E1A exemplifies this through its association with cellular DCAF7 (DDB1 and CUL4-associated factor 7) (65) (Fig. 1). DCAF7 contains a WD40 domain, which offers a surface for several protein-protein interactions and is a feature of many cellular scaffolds (66). DCAF7 directly binds and coordinates multiple kinases involved in transcriptional regulation, cell proliferation, and differentiation, including dual-specificity tyrosine-regulated kinases (DYRK1A and DYRK1B) and homeodomain-interacting protein kinase 2 (HIPK2) (67, 68). DCAF7 therefore also acts as a bridge between HAdV E1A and these kinases, enabling viral manipulation of their localization and catalytic activities.

E1A’s effects on DYRK1A via indirect association have been well characterized. These include stimulating its kinase activity *in vitro* as well as utilizing it for E1A-mediated transformation of mammalian cells (53, 69). The E1A-HIPK2 interaction was more recently reported and it remains to be seen if it functions analogously to DYRK1A. Additionally, HAdV-5 E1A acts as a substrate for both kinases (on Ser-231) which enables a downstream interaction between the FOXK1/2 transcription factors (TFs) and E1A’s SSTDSCDSGPS SLiM (65) (Fig. 2). While the interaction with FOXK1/2 appears specific for E1A from HAdV-C, the same SLiM is present in the E6 proteins of some cutaneous HPVs (70).

E1A was also previously reported to associate with a receptor for activated C kinase 1 (RACK1), another WD-containing scaffold protein (71). While this interaction remains largely uncharacterized, other viral proteins have been shown to manipulate RACK1 functions to gain access to the protein kinase C apparatus (72–76). Given the pervasiveness of these strategies among distinct viruses, discoveries of manipulation of scaffolds and adapters will undoubtedly continue.

### Mimicry of A-kinase anchoring proteins.

The highly organized nature of cells requires orchestration of countless protein-protein interactions mediated via structural domains or via motifs such as SLiMs. This can be exploited by viruses such as HAdV, whose enhanced evolutionary flexibility allows them to rapidly acquire new and useful interaction motifs as adaptive responses to differing cellular environments. This can enable integration into (and rewiring of) host networks using protein interaction motifs that mimic cellular counterparts (77–80).

HAdV E1A illustrates the effectiveness of viral mimicry through its interaction with PKA (80–82) (Fig. 1). PKA is a well-studied and highly conserved kinase functioning in a variety of host processes, including growth and cellular gene expression (64, 83). PKA is a tetramer composed of a regulatory subunit dimer bound to two catalytic subunits; these dissociate when the second messenger, cAMP, binds and activates the holoenzyme. A-kinase anchoring proteins (AKAPs) spatially and temporally direct PKA function via binding the regulatory subunits using a well-studied amphipathic α-helix motif (62–64). HAdV E1A was found to also contain an AKAP-like helix in its sequence, and this was validated to confer association with PKA on the same molecular sites as cellular AKAPs (80, 81) (Fig. 2). This mimicry allowed E1A to outcompete endogenous cellular AKAPs for PKA binding during HAdV infection and relocalize PKA subunits to the nucleus. Consequently, E1A’s ability to act as a “viral AKAP” contributed to its transactivation functions and to cooperate with the cell’s cAMP signaling pathway. This enhanced multiple aspects of the viral replication cycle, including transcription, protein synthesis, genome replication, and progeny production. The mechanism was also conserved in E1A proteins across several species of HAdV, indicating its evolutionary importance (82). It remains to be seen if the presence or absence of AKAP mimicry by E1As from different HAdV species contributes to the various tropisms or pathogeneses in different HAdVs. Given PKA’s critical role in cell biology and consequent link to cellular diseases when dysregulated (84), there may be other viruses with proteins capable of either hijacking or mimicking cellular AKAPs like E1A.

Instead of encoding entire orthologs of large cellular components or evolving complicated globular structures, mimicry provides an efficient means of increasing the functionality of the size-constrained HAdV genome. The success of viral mimicry is demonstrated by how often it has evolved convergently in genetically distinct viruses (85–87). It is also telling that virtually all cellular processes have been shown to be susceptible to viral “corruption” in this manner (1). Understanding viral hijacking of host components via mimicry reveals new molecular aspects of the relationships between cell regulation, viral pathogenesis, and human disease.

## DATA MODIFICATION

No virus is capable of encoding all the machinery required to independently produce its own proteins and metabolic energy. Many viruses also require additional enzymes to produce nucleic acids for genomes or transcripts. HAdV requires host RNA polymerases and DNA-binding transcription factors (TFs) in order to transcribe its own genes (88). The HAdV genome has evolved to contain many *cis*-acting elements capable of engaging with RNA polymerase II (RNAPII) and various TFs, but the coordination of these virus-host interactions is multifactorial and complex (89–97). E1A’s ability to act as a molecular hub is crucial in the context of transcriptional control. It can associate with various eukaryotic transcriptional components and modify their activities to modulate viral gene expression (Fig. 1).

### Hijacking general transcriptional machinery.

E1A activates transcription of the other HAdV early genes, which encode components that perform crucial tasks during infection, including inhibiting apoptosis, replicating the viral genome, suppressing immune responses, and transporting viral mRNA (98–101). While the transactivation ability of E1A largely maps to its N terminus and CR3 regions (both can drive reporter gene expression when fused to a heterologous DNA-binding domain) (102), E1A makes protein-protein interactions along its entire sequence that contribute to transcriptional regulation, exemplifying its modular nature.

Transactivation by E1A has been best characterized within its CR3 region, one of the few E1A portions containing structure (a zinc finger) (103–105) (Fig. 2). Here, E1A binds and recruits several components of the TFIID complex to nucleate formation of the RNAPII preinitiation complex (106–109) (Fig. 1). These interactions alone are not sufficient for maximal levels of E1A-mediated transactivation, which also requires MED23, a component of the multiprotein Mediator adapter complex. Its recruitment to promoters serves as a potent activation step (110–112). While E1A lacks direct DNA-binding ability, it does bind to several promoter-targeting TFs, including members of the cAMP response element/activating transcription factor (ATF) family, activator protein-1 (AP-1), upstream stimulatory factor (USF), and Sp1 (113–115). Additionally, E1A CR3 binds coactivators (such as pCAF and p300) or even repressors of transactivation, including GCN5 and BS69 (116–118). Therefore, this portion of E1A appears to coordinate the presence of sequence-specific TFs with both activating and repressive complexes, allowing for tight control of viral transcription kinetics.

Mechanisms of transcriptional regulation via other regions of E1A are less well understood; however, cellular targets of both the N- and C-terminal portions of E1A contribute to the full extent of this function. CtBP1 and CtBP2 are bound by the PxDLS SLiM in E1A’s C terminus and normally function as transcriptional corepressors (52, 119) (Fig. 2). E1A competes with endogenous proteins to bind CtBP1/2, relieving repression of host genes involved in cell growth and apoptosis (120, 121). E1A also uses CtBP1/2 to bring itself to and activate promoters that are normally repressed (effectively converting cellular repressors into coactivators) (122). This region also binds the sequence-specific TFs FOXK1 and FOXK2 (65, 70), and exciting new results from the Berk laboratory demonstrate that single molecules of E1A must simultaneously form complexes with FOXK1/2, DCAF7, and CtBP during infection in order to suppress IRF3-driven activation of interferon (IFN)-stimulated genes (ISGs) (123). These findings provided precise molecular details of how E1A acts as a molecular scaffold to carry out this newly discovered function of its C terminus. Separately, a comprehensive analysis of C-terminal mutants carried out by the Pelka laboratory showed that deletions outside the regions with known binding partners also impact gene expression and virus replication—suggesting that novel binding partners of E1A’s C terminus still remain to be discovered (124).

The N terminus of E1A is densely packed with many overlapping protein-protein interaction sites. These include SLiMs conferring interactions with TFs/cofactors such as p300/CBP, myogenin, AP-2, p400, PKA, Bre1, TRRAP, thyroid hormone receptor (TR), and components of the proteasome (80, 125–135) (Fig. 1 and 2). It is still not entirely clear how some of these interactions benefit HAdV infection. However, different regions of E1A have been shown to cooperate to maximize control over specific interaction partners or to establish novel connections that regulate viral gene expression, as highlighted above (123, 136, 137). Effectively, E1A functions as a viral hub to coordinate a massive reshuffling of host transcriptional machinery. This is exemplified in reports showing that expression of virtually every cellular gene in infected cells is altered in an E1A-dependent fashion (25, 26, 138, 139).

### E1A enhances transcriptional elongation.

Research on E1A-dependent activation of viral gene expression has largely focused on the initiation step of transcription. However, transcription is a multistage process also involving the regulation of elongation and termination (140). Recent studies on E1A revealed that it also enhances elongation of HAdV transcripts produced by RNAPII. The potential for E1A to enhance elongation was established from observations that cellular factors recruited to promoters in an E1A-dependent manner (and E1A itself) were also found associated with downstream areas being actively transcribed (141). These initial findings were expanded upon in studies examining E1A’s direct interaction with MED23. MED23 bridged associations between E1A and other components of the Mediator complex, including MED26 and MED26-associated super elongation complex (SEC) (142). These interactions all contributed to E1A-mediated activation of HAdV-5 early gene expression. However, the SEC also contains factors that regulate the elongation of transcripts produced by RNAPII. Specifically, the SEC can relieve pausing of RNAPII on nascent mRNA, which serves as a form of checkpoint control (143). While a specific role for the SEC in the elongation of HAdV transcripts was not elucidated, these findings demonstrated that E1A-mediated transactivation was likely more complex than the mere triggering of efficient formation of preinitiation complexes.

Subsequent investigation into elongation effects conferred by E1A resulted in the discovery of a bona fide mechanism driving elongation of HAdV transcripts (144). E1A associated indirectly with the human Paf1 complex, which is composed of several proteins that accompany RNAPII from the promoter to the 3′ end of mRNA. In this capacity, Paf1 participates in multiple aspects of transcription, including recruitment of histone modifying enzymes, assembly of elongation factors that prevent dissociation of RNAPII from the template, and association with factors required for proper termination of newly synthesized mRNA (145, 146). Loss of an E1A-Paf1 interaction during HAdV infection did not noticeably affect the initiation of HAdV early gene expression; however, there were drastic decreases in the levels of both E1A and RNAPII occupancy at the 3′ ends of early transcription units, implying severe defects in the elongation efficiency of these transcripts. This was confirmed by the detection of decreased frequencies of both full-length transcripts and H3K36 trimethylation (an elongation-specific chromatin mark) under these conditions (144, 147).

The aforementioned studies established an enhanced model of E1A-mediated viral transcription that includes both initiation- and elongation-specific functions. It is unknown if E1A interacts with components of other complexes involved in transcriptional elongation or if E1A affects elongation of eukaryotic transcripts. While other human pathogens such as influenza A virus, human immunodeficiency virus, and herpes simplex virus have been previously shown to manipulate elongation in various ways, it remains to be determined whether E1As from all HAdV species behave similarly (148–150).

## INSTALLING TOOLS

The proteome of a human cell is extensively regulated by posttranslational modifications (PTMs). HAdV E1A can cause extensive reorganization of PTMs by sterically inhibiting host enzymes or acting as a bridge between an enzyme and substrate to establish new interactions. Large-scale studies on E1A-mediated modifications of host proteins have revealed how drastically the cellular proteome is altered during HAdV infection (25–27, 137, 139). Consequently, how pathogens such as HAdV repurpose endogenous enzymes to deregulate host epigenetics, signaling pathways, and protein localization or stability remains an expanding area of molecular research.

### Remodeling chromatin posttranslational modifications.

Next-generation sequencing technology revealed that E1A-mediated gene regulation includes global effects on host cell transcription. During HAdV-5 infection of primary cells, E1A associates with the regulatory regions of more than 17,000 host genes (25, 27), massively altering cellular transcription (15, 26, 138). Histone PTMs (hPTM) offer an additional level of host genetic regulation by allowing or restricting access of transcriptional machinery to chromatin, a mechanism extensively modulated by E1A during infection (25, 139, 151). These changes in chromatin structure are caused by E1A binding to and altering the functions of numerous chromatin-regulating factors, including p300/CBP, PCAF, Bre1, GCN5, p400, and BS69 (Fig. 1). For example, E1A’s concomitant associations with lysine acetyl transferases (KAT), p300 and CBP, and thousands of host gene promoters result in widespread changes in acetylation of H3K18 across the human genome with retargeting of these enzymes to specific genomic loci (25, 27). Consequently, many cellular genes involved in differentiation or innate immunity become transcriptionally inactive, while those involved in cell cycle and macromolecular synthesis are upregulated, creating a cellular environment conducive to HAdV replication.

More-recent studies revealed multiple epigenetic mechanisms of E1A-mediated suppression of host IFN responses. First, E1A’s interaction with Bre1 (an E3 ubiquitin ligase involved in modifying histone H2B) blocked Bre1’s ability to monoubiquitinate H2B during HAdV infection (128). This interfered with RNAPII’s ability to efficiently transcribe from these loci, resulting in repression of the ISGs that require this specific hPTM. Second, the C terminus of E1A was shown to bind RuvBL1 (an ATP-dependent DNA helicase and a component of the NuA4 HAT complex) (152, 153). During HAdV infection, E1A and RuvBL1 colocalized to IFN-sensitive response elements, repressing expression of RuvBL1-regulated ISGs (though the mechanism remains unknown). Together, these separate mechanisms reinforce the idea of the critical need for HAdV to downregulate its host’s IFN response.

Additional chromatin regulatory factors that directly interact with E1A include BS69, GCN5, TRRAP, and p400. BS69 is a multidomain protein that acts as an inhibitor of E1A transactivation (117). It binds directly via a conserved PxLxP SLiM present in E1A CR2 (29, 154) (Fig. 2). Recently, BS69 was shown to repress expression of genes decorated with histone H3.3 trimethylated at K36 by increasing intron retention and abrogating RNAPII elongation (155, 156). The effects of E1A on BS69-mediated chromatin regulation during infection are largely uncharacterized, warranting further investigation. GCN5 is a histone KAT that binds directly to multiple parts of E1A, including the N terminus and CR3 transactivation domains (116). Although the GCN5-E1A interaction has been studied in the context of HAdV replication and gene expression, E1A-mediated modulation of its KAT activity has yet to be examined across the cellular transcriptome. Related E1A partners include TRRAP and p400, participating along with GCN5 and PCAF in multiprotein histone acetyltransferase activation (HAT) complexes (129, 157). Interaction with TRRAP and p400 promotes the association of MYC with the NuA4 complex (126, 158). This was recently shown to upregulate a unique panel of MYC-associated genes involved in gene regulation and ribosome biogenesis (159). Furthermore, p400 was recently found to play a role in the deposition of histone H2AZ and H3.3 at transcriptionally active genes (160), opening another unexplored avenue of gene modulation by E1A.

E1A’s transcriptional reprogramming of the host blocks innate immunity, induces the cell cycle, and reverses the differentiated phenotype in quiescent cells (17, 20). This provides abundant precursor pools of deoxynucleoside triphosphates (dNTPs) and NTPs for viral DNA and RNA synthesis and activates pathways required for protein synthesis. E1A also represses antiviral response genes that normally inhibit virus replication and spread. The sum of these effects is a host environment optimal for a productive HAdV replication cycle. How E1A repurposes endogenous chromatin remodeling machinery to alter downstream pathways remains an active area of molecular research.

### Rewiring SUMOylation pathways.

In humans, the small ubiquitin-like modifier (SUMO) proteins are a family of proteins composed of SUMO1 through SUMO4 (161–163). Posttranslational covalent attachment of these moieties to lysine residues in target proteins is referred to as SUMOylation. This process is linked to various cellular processes in the nucleus, including nuclear transport, DNA replication, and gene expression. Conjugation of SUMO is similar to ubiquitination—SUMO precursors are processed by proteases before activation by E1-activating enzymes and transferred to target proteins by the E2-conjugating enzyme (UBC9) alone or in conjunction with the E3-protein ligase. Because SUMOylation of proteins typically affects nuclear processes, it is unsurprising that the predominantly nuclear E1A protein hijacks components of this PTM cascade.

UBC9 is a known binding target of E1A (164). The N-terminal portion of UBC9 binds to the EVIDLT SLiM in CR2 of HAdV-5 and HAdV-12 E1As (165) (Fig. 2). This interferes with UBC9-mediated polySUMOylation, resulting in reorganization of PML nuclear bodies by E1A (166). PML is an antiviral TRIM protein, and PML nuclear bodies are routinely found to be altered during infection by viruses that replicate in the nucleus (167, 168). Clearly, these subcellular loci form a crucial battleground between pathogen and host which can be probed at the molecular level using tools such as E1A. For a long time, the only host protein whose SUMOylation was known to be affected by E1A was pRB (169). However, E1A’s interaction with UBC9 was recently shown to affect SUMOylation of DREF (170). DREF is one of the growing number of host proteins that directly bind the C terminus of E1A. DREF can localize to HAdV replication centers and restrict viral replication; to counteract this function, E1A enhances SUMOylation and subsequent relocalization of DREF within PML nuclear bodies.

Subcellular redistribution of cellular enzymes responsible for the addition or subtraction of function-altering moieties is a common theme in viral infections. HAdV is no exception and employs E1A to contribute to an extensive reorganization of PTMs within infected cells. Histones, TFs, and cell cycle regulators are just some of the critical targets of this E1A function and comprise an already impressive list that will only expand as genome-wide studies involving E1A are completed.

## DENIAL OF SERVICE ATTACK

While HAdV E1A can massively alter the transcriptional profile of host cells, there are additional ways of regulating viral and cellular protein levels during infection (1). In eukaryotes, the 26S proteasome represents the major nonlysosomal proteolytic machinery serving to degrade targeted proteins in an ATP-dependent manner (171, 172). The proteasome is a multiprotein unit composed of a regulatory complex, proteolytic core, and several homologous ATPases. Polyubiquitination of proteins typically targets them for degradation by the proteasome. Ubiquitination requires a multienzyme cascade involving a ubiquitin activator (E1), ubiquitin conjugator (E2), and ubiquitin ligase (E3). Hundreds of variants of E3 ligases exist and can apply a diversity of tags to targeted proteins. By attacking the ubiquitin-proteasome cascade, viruses can deny the cell access to this crucial function and promote longer or shorter half-lives of selected protein substrates.

### Fine-tuning ubiquitination.

The Skp, Cullin, F-box-containing complex (SCF) is a multicomponent ubiquitin ligase containing several invariant components and a variable F-box protein that functions as the substrate receptor (173). SCF complexes are targeted for modulation by HAdV E1A, and (depending on the associated F-box protein) this results in either upregulation or downregulation of the destruction of SCF-recognized targets (174, 175). E1A binds directly to a complex containing the F-box protein, Fbw7 (174). This interaction inhibited catalysis of ubiquitin chain formation *in vitro*. While the inhibitory mechanism was not fully unraveled, the result was a decrease in the levels of ubiquitinated forms of several Fbw7-recognized cellular targets and an increase in their stabilities. These included oncogenic proteins c-Myb, c-Jun, cyclin E, and MYC, the attenuated turnover of which suggests their involvement in E1A-mediated promotion of cell proliferation.

Conversely, E1A can also encourage degradation of SCF targets by upregulating a different F-box protein, β-TrCP (175). Rather than using a direct interaction with this complex, E1A upregulates β-TrCP protein through an undefined mechanism. This upregulation enhanced the degradation of repressor element 1-silencing TF (REST), a tumor suppressor implicated in regulating over 1,000 cellular genes, many involved in cell proliferation and migration (176). E1A is thought to relieve repression of REST-modulated genes by inducing ubiquitination and proteasomal degradation via β-TrCP, leading to increased cell proliferation and anchorage-independent growth.

E1A’s ability to selectively inhibit or induce degradation of subsets of host proteins by differentially affecting ubiquitin ligases demonstrates an elegant, bidirectional ability to fine-tune cellular protein kinetics. While the studies discussed here suggested roles contributing to E1A’s oncogenic functions, many mechanistic details remain unclear. The potential for other types of E3 ligases to be affected by E1A to enhance the HAdV replication cycle and E1A-mediated transformation is an unexplored possibility.

### Interacting with various proteasomal subunits.

Downstream of ubiquitin ligase complexes, the multisubunit 26S proteasome carries out proteolysis of proteins targeted for degradation (171). HAdV E1A interacts with proteasomal subunits in both the cytoplasm and nucleus. These include parts of both the 20S proteolytic core and 19S regulatory component (S2, S4, and S8) mediated via multiple binding sites on E1A (134, 141). Fascinatingly, these proteins were recruited to HAdV early gene promoters in an E1A-dependent manner and were necessary for efficient transcriptional initiation from these sites (141). In a tradeoff, the E1A-proteasome interactions decreased the half-life of E1A while enhancing overall viral gene expression, also revealing a new function for the repurposing of this host pathway which may play a role in promoter clearance by RNAPII (177). Aside from transcriptional regulation, E1A’s direct interactions with the proteasome may also enable control of host protein abundance during infection. This was indicated by observations revealing that E1A inhibited the ATPase activity of both S4 and S8 *in vitro*, suggesting that E1A may block degradation of proteins by the 26S proteasome. This was later confirmed *in vivo*, where all of E1A’s interactions with S2, S4, and S8 contributed to prolonging the half-life of the proteasome target (p53) irrespective of its ubiquitination status (134). Thus, it remains plausible that E1A extends the life spans of other cellular proteins that would normally be quickly degraded.

In addition to general degradation of targeted proteins, the proteasome participates in production of peptides for major histocompatibility complex (MHC) class I presentation (178). This essential part of immune surveillance is a pathway that has been targeted for disruption by numerous viruses. In response to proinflammatory signals, alternative subunits of the immunoproteasome can be expressed and can substitute for the more generic counterparts (179). These include 20S proteasome subunit β-2i (PSMB10), a direct binding partner of E1A (180). It is speculated that E1A binding to the immunoproteasome may inhibit its ability to process antigens and produce epitopes for immune recognition. Reinforcing this inhibitory hypothesis, it appears that E1A has evolved a second, unrelated mechanism for downregulating this component of the immunoproteasome—specifically by reducing its expression in the presence or absence of gamma interferon (IFN-γ). Currently, the detailed mechanisms and *in vivo* consequences for E1A-mediated disruption of protein processing via the immunoproteasome remain to be investigated.

## CORRUPTING THE HOST SYSTEM

Radical alterations to cellular protein interaction networks are seen not only during infections by DNA tumor viruses but also in many types of cancer. Given that the functions of viral oncoproteins such as E1A and the *cis*-acting genome variations found in tumors often converge on the same pathways, E1A is a valuable tool for identifying host proteins affecting transformation and tumorigenesis. In fact, analyses using *trans*-acting viral products have been demonstrated to be capable of successfully predicting cancer-relevant targets at rates on par with functional genomics and large-scale cataloging of tumor mutations (5).

### Controlling the cell cycle.

HAdV does not cause cancer in humans, possibly because lytic infection is typically rapid and self-limiting and because the viral genome does not normally integrate into the host’s (181–183). However, E1A alone can immortalize cells in culture when introduced by stable transfection. In combination with a cooperating oncogene, such as HAdV E1B or activated Ras, it can fully transform these cells (16, 19).

E1A’s assault on cell cycle regulators involves crucial interactions with retinoblastoma protein pRB (RB1) and related family member proteins p107 (RBL1) and p130 (RBL2) (17) (Fig. 1). In growth-arrested cells, hypophosphorylated pRB controls cell proliferation by complexing with members of the E2F family of TFs, interfering with their ability to activate transcription of genes required for S-phase induction. Under normal conditions of cellular control, phosphorylation of pRB by cyclin-dependent kinases releases E2F from pRB, allowing E2F-dependent transactivation. During HAdV infection, E1A binds hypophosphorylated pRb to free E2F, overriding this proliferative control mechanism. In addition to pRB, E1A can also associate directly with promoter-bound E2F/DP-1 complexes to activate cell cycle genes (184). The pRb-E1A interaction is mediated primarily through an LxCxE SLiM found in CR2 and through a second site in CR1 (29, 185, 186) (Fig. 2). The LxCxE SLiM has recently been shown to also confer an interaction with STING, suggesting a new role for E1A in antagonizing the sensing by the cell of viral DNA (187). Furthermore, E1A proteins can form trimeric complexes between pRB and p300/CBP, causing acetylation of pRB and contributing to potent oncogenic activity in primary cells (137).

Recently, novel E1A interacting partners have been shown to contribute to this deregulation of cell cycle control. E1A binds to NimA-related protein kinase 9 (Nek9) through its N terminus (188, 189). Nek9 has a role in mitosis (required for proper centrosome separation) and is involved in the DNA replication stress response (190, 191). Nek9 colocalized with E1A at the p53-regulated GADD45A (growth arrest and DNA-damage-inducible, 45 alpha) internal promoter. This caused transcriptional suppression of GADD45A, whose protein products are important in cell division and DNA damage-induced cell cycle arrest (192). High levels of GADD45A also hinder viral genome replication, and the E1A-Nek9-mediated repression enhanced viral progeny production. Ku70 (XRCC6) was also recently described as a binding target of E1A’s C terminus (193). Ku70 is an abundant protein that plays a role in the double-strand DNA break repair pathway in mammals (194). It participates in preservation of genome integrity, telomere maintenance, and inhibition of apoptosis via sequestration of Bax from the mitochondria. E1A caused Ku70’s relocalization to viral replication centers and association with the HAdV genome. Ku70 was also found to be recruited to host cell cycle-regulated promoters during infection, but how it was recruited and for what purpose remain unknown. Importantly, depletion of Ku70 was associated with reduced viral yields and an impaired ability of HAdV to drive quiescent cells into S phase.

Collectively, these findings illustrate the diverse means by which E1A ensures tight control over the cell cycle. Under normal infection conditions, this benefits HAdV; in isolation, however, E1A’s oncogenic properties can be used to enhance knowledge of fundamental processes leading to cancer.

## CONCLUSION

Currently, dozens of distinct cellular protein targets are reported to directly bind HAdV E1A, and the potential secondary interactions extend to thousands of other cellular targets (Fig. 1). These interactions alter their normal cellular functions and localizations and establish new molecular connections within the cell’s protein network. The wide diversity of E1A’s targets provides to HAdV an enormous amount of control over viral and cellular gene expression, allowing reprogramming of virtually all cellular processes. E1A’s highly modular nature has also made it ideal for mutagenesis studies. The identification of its many binding partners, along with characterization of the interaction motifs involved, has provided tremendous insight into eukaryotic protein biology (Fig. 2). Indeed, the biological roles of many host regulatory proteins and their places within the cellular protein interaction network were revealed as a result of having been identified originally as E1A binding partners. While most studies of E1A functions have focused on the E1As of highly related types HAdV-2 and HAdV-5, seven distinct species comprising over 50 unique types of HAdV are known to exist. The differences between the sequences of these E1As, their interaction partners, and their functions may provide insight into the evolutionary history of HAdVs as well as reveal molecular details of their various tropisms and pathogeneses. Therefore, by continuing to use genetic and biochemical approaches to discover novel interacting partners of E1A, we may identify as-yet-unknown targets important in various cellular processes.
